# Supplementary material for: Ideal acoustic quantum spin Hall phase in a multi-topology platform
Source: Nat Commun. 2023 Feb 20;14:952. doi: 10.1038/s41467-023-36511-2 (PMC9941186; doi:10.1038/s41467-023-36511-2)
Supplement: Supplementary file 1 — Supplementary Information for [file 41467_2023_36511_MOESM1_ESM.pdf]

Supplementary Information for **"Ideal acoustic quantum spin Hall phase in a multi-topology platform"**

Xiao-Chen Sun[1,2,§], Hao Chen[1,§], Hua-Shan Lai[1,§], Chu-Hao Xia[1], Cheng He[1,2,3,*], Yan-Feng Chen,[1,2,3,*]

[1]National Laboratory of Solid State Microstructures & Department of Materials Science and Engineering, Nanjing University, Nanjing 210093, China

[2]Collaborative Innovation Center of Advanced Microstructures, Nanjing University, Nanjing 210093, China

[3]Jiangsu Key Laboratory of Artificial Functional Materials, Nanjing University, Nanjing 210093, China

*Corresponding author.

chenghe@nju.edu.cn

yfchen@nju.edu.cn

§X.-C. S., H. C., H.-S. L., contributed equally to this work.

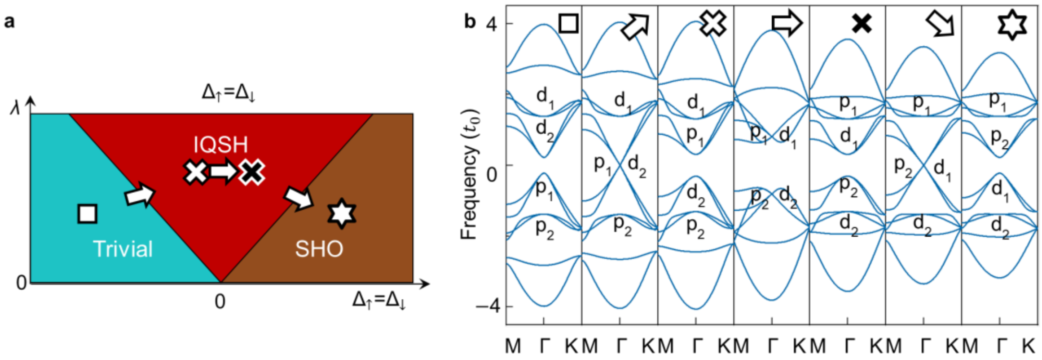

Fig. S1 | Bulk bands of different phases from tight-binding approximation (TBA). a. Phase transition diagram in the $\Delta_\uparrow = \Delta_\downarrow \equiv \Delta$ vertical section. **b**. Bands of different phases from TBA. The square indicates the trivial phase with $\lambda = 0.58$ and $\Delta = -0.8$. The arrow pointing to higher right indicates the phase transition from the trivial to IQSH phases with $\lambda = 0.7$ and $\Delta = -0.7$. The hollow fork indicates IQSH phase with $\lambda = 0.8$ and $\Delta = -0.5$. The arrow pointing to right still indicates IQSH phase with $\lambda = 0.8$ and $\Delta = 0$ with no phase transition, although there is a band inversion between $|p\rangle_{2,1}$ and $|d\rangle_{1,2}$ states. The solid fork still indicates IQSH phase with $\lambda = 0.8$ and $\Delta = 0.5$. The arrow pointing to lower right indicates the phase transition from IQSH to SHO phases with $\lambda = 0.7$ and $\Delta = 0.7$. The hexagram indicates SHO phase with $\lambda = 0.58$ and $\Delta = 0.8$. The unit of frequency is set to be $t_0 = 1$.

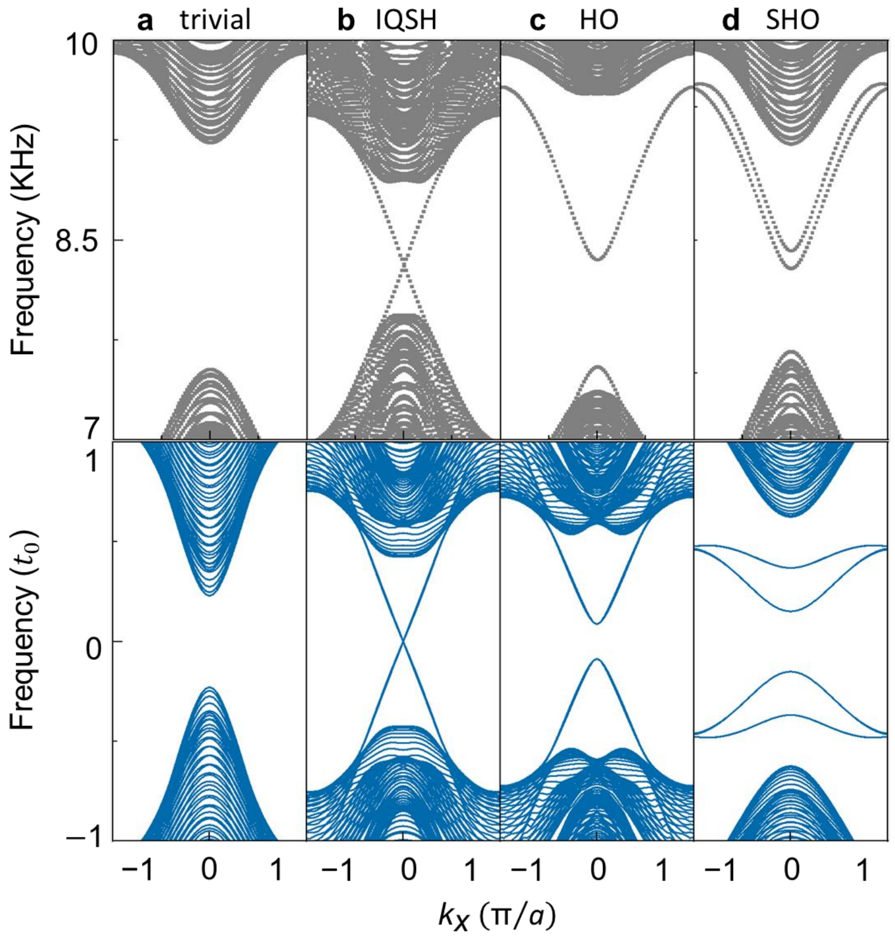

Fig. S2 | Dispersion relations of super unit cell (UC) for trivial, IQSH, HO and SHO phases from simulation and TBA, respectively. a. Trivial phase. $w_{\mathrm{o}\uparrow\downarrow} = 0.13\mathrm{cm}$, $w_{\mathrm{i}\uparrow\downarrow} = 0.33\mathrm{cm}$ and $R_\lambda = 0.2a/6$ for simulation. $\Delta_\uparrow = \Delta_\downarrow = -0.8$ and $\lambda = 0.58$ for TBA. **b**. IQSH phase. $w_{\mathrm{o}\uparrow\downarrow} = 0.29\mathrm{cm}$, $w_{\mathrm{i}\uparrow\downarrow} = 0.31\mathrm{cm}$, and $R_\lambda = 0.35a/6$ for simulation. $\Delta_\uparrow = \Delta_\downarrow = -0.2$ and $\lambda = 0.8$ for TBA. **c**. HO phase. $w_{\mathrm{o}\uparrow} = 0.4\mathrm{cm}$, $w_{\mathrm{o}\downarrow} = 0.13\mathrm{cm}$, $w_{\mathrm{i}\uparrow} = 0.2\mathrm{cm}$, $w_{\mathrm{i}\downarrow} = 0.33\mathrm{cm}$, and $R_\lambda = 0.2a/6$ for simulation. $\Delta_\uparrow = -\Delta_\downarrow = -0.2$ and $\lambda = 0.8$ for TBA. **d**. IQSH phase. $w_{\mathrm{o}\uparrow\downarrow} = 0.4\mathrm{cm}$, $w_{\mathrm{i}\uparrow\downarrow} = 0.2\mathrm{cm}$ and $R_\lambda = 0.2a/6$ for simulation. $\Delta_\uparrow = \Delta_\downarrow = 1.2$ and $\lambda = 0.58$ for TBA.

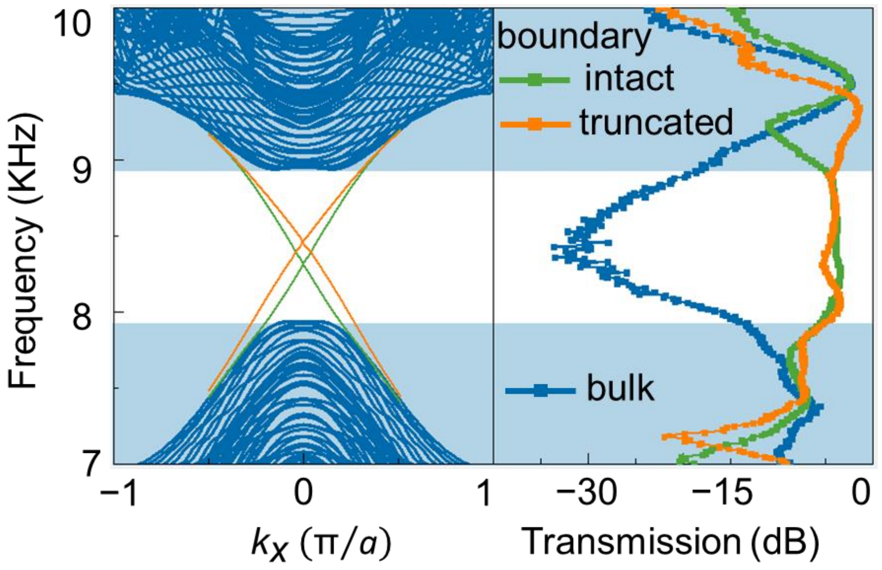

Fig. S3 | Numerical projected band structures and experimental transmission spectra for intact and truncated boundary in IQSH phase, respectively. Blue, green and orange lines represent states and transmission for bulk, intact boundary and truncated boundary, respectively.

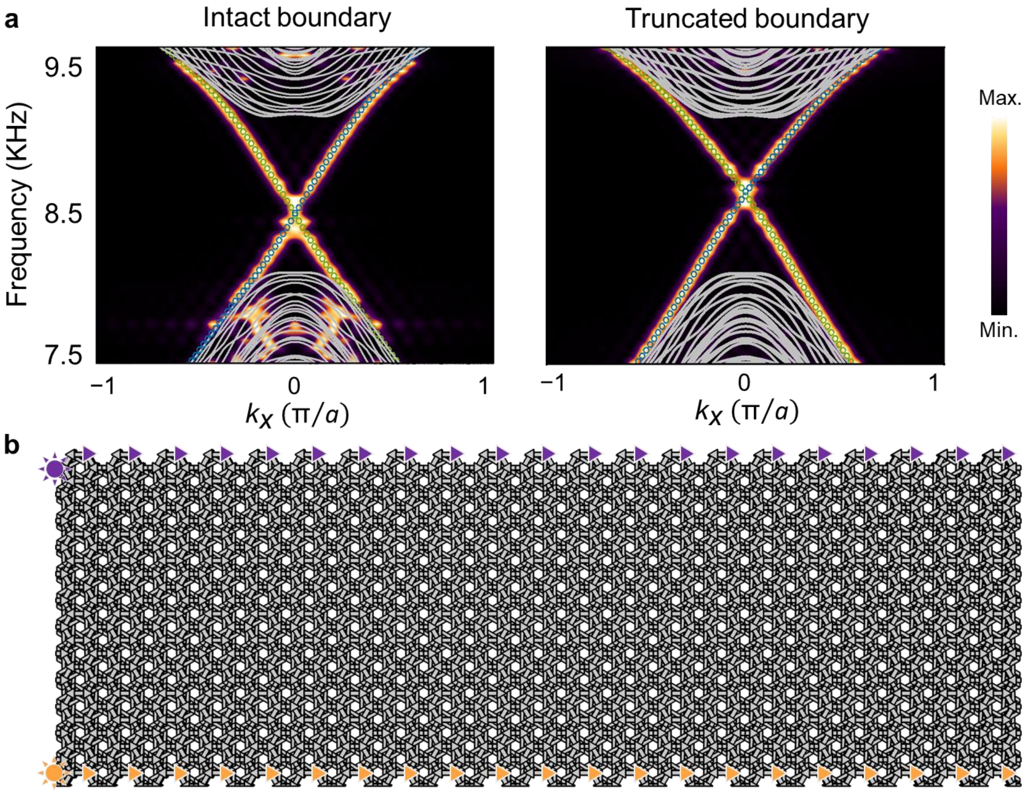

Fig. S4 | Dispersion of helical edge states on the truncated boundary. a. Simulated band structure with intact (purple) and the truncated (Orange) boundary. Color scale represents the density of acoustic energy density from simulation. Gray lines and Blue (green) circles denote bulk and edge states calculated from super-UCs shown in the right panel. **b.** The sample in the simulation. The source is marked as the purple suns. The probes to collect acoustic pressures are set on sites marked by yellow triangles. We calculate the stationary states with frequency from 7.5KHz to 9.5KHz and process data with Fourier transform to get the dispersion results.

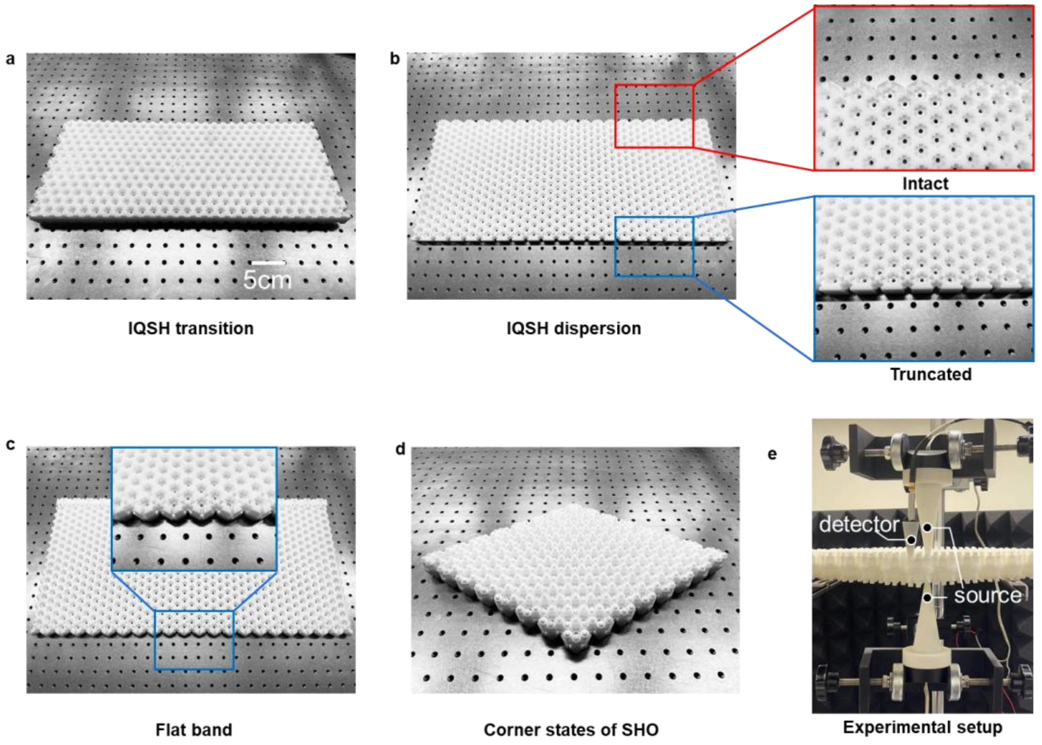

Fig. S5 | Samples in experiments. a. Experimental samples to measure transmission spectrum for the IQSH phase. **b**. Experimental samples with intact, truncated boundary in the IQSH phase. **c**. Experimental samples to measure once-folded flat band. **d**. Experimental samples to measure the response for corner states in the SHO phase. **e**. Experimental setup to measure symmetry and anti-symmetry corner states in the SHO phase.

I. Kane-Mele-like tight-binding model

As Eq. (1) in the main text, the Hamiltonian is

$$H = -\sum_{\langle m,n\rangle_{\mathrm{o}}\alpha} t_{\mathrm{o}\alpha}\,\hat{c}_{m\alpha}^{\dagger}\hat{c}_{n\alpha} - \sum_{\langle m,n\rangle_{\mathrm{i}}\alpha} t_{\mathrm{i}\alpha}\,\hat{c}_{m\alpha}^{\dagger}\hat{c}_{n\alpha} - \frac{\lambda}{3}\sum_{\langle\langle m,n\rangle\rangle,\alpha\neq\beta} v_{mn,\alpha}\hat{c}_{m\alpha}^{\dagger}\hat{c}_{n\beta} \tag{S1}$$

Expanding this Hamiltonian in momentum space and dividing it into two parts with the basis defining on sites $(|1\rangle_\alpha, |2\rangle_\alpha, |3\rangle_\alpha, |4\rangle_\alpha, |5\rangle_\alpha, |6\rangle_\alpha)^T$, $\alpha = \uparrow\downarrow$, respectively. We can get

$$H = \begin{pmatrix} H_{\uparrow\uparrow} & H_{\uparrow\downarrow} \\ H_{\uparrow\downarrow}^{\dagger} & H_{\downarrow\downarrow} \end{pmatrix} \tag{S2}$$

, where

$$H_{\alpha\alpha} = \begin{bmatrix} 0 & -t_{\mathrm{i}\alpha} & 0 & -t_{\mathrm{o}\alpha}e^{-i\boldsymbol{k}\cdot\boldsymbol{a_1}} & 0 & -t_{\mathrm{i}\alpha} \\ -t_{\mathrm{i}\alpha} & 0 & -t_{\mathrm{i}\alpha} & 0 & -t_{\mathrm{o}\alpha}e^{i\boldsymbol{k}\cdot\boldsymbol{a_2}} & 0 \\ 0 & -t_{\mathrm{i}\alpha} & 0 & -t_{\mathrm{i}\alpha} & 0 & -t_{\mathrm{o}\alpha}e^{-i\boldsymbol{k}\cdot\boldsymbol{a_3}} \\ -t_{\mathrm{o}\alpha}e^{i\boldsymbol{k}\cdot\boldsymbol{a_1}} & 0 & -t_{\mathrm{i}\alpha} & 0 & -t_{\mathrm{i}\alpha} & 0 \\ 0 & -t_{\mathrm{o}\alpha}e^{-i\boldsymbol{k}\cdot\boldsymbol{a_2}} & 0 & -t_{\mathrm{i}\alpha} & 0 & -t_{\mathrm{i}\alpha} \\ -t_{\mathrm{i}\alpha} & 0 & -t_{\mathrm{o}\alpha}e^{i\boldsymbol{k}\cdot\boldsymbol{a_3}} & 0 & -t_{\mathrm{i}\alpha} & 0 \end{bmatrix} \tag{S3}$$

$$H_{\uparrow\downarrow} = \frac{\lambda}{3}\begin{bmatrix} 0 & 0 & -1-e^{-i\boldsymbol{k}\cdot\boldsymbol{a}_1}-e^{i\boldsymbol{k}\cdot\boldsymbol{a}_3} & 0 & 0 & 0 \\ 0 & 0 & 0 & -1-e^{-i\boldsymbol{k}\cdot\boldsymbol{a}_1}-e^{i\boldsymbol{k}\cdot\boldsymbol{a}_2} & 0 & 0 \\ 0 & 0 & 0 & 0 & -1-e^{-i\boldsymbol{k}\cdot\boldsymbol{a}_3}-e^{i\boldsymbol{k}\cdot\boldsymbol{a}_2} & 0 \\ 0 & 0 & 0 & 0 & 0 & -1-e^{i\boldsymbol{k}\cdot\boldsymbol{a}_1}-e^{-i\boldsymbol{k}\cdot\boldsymbol{a}_3} \\ -1-e^{-i\boldsymbol{k}\cdot\boldsymbol{a}_2}-e^{i\boldsymbol{k}\cdot\boldsymbol{a}_1} & 0 & 0 & 0 & 0 & 0 \\ 0 & -1-e^{-i\boldsymbol{k}\cdot\boldsymbol{a}_2}-e^{i\boldsymbol{k}\cdot\boldsymbol{a}_3} & 0 & 0 & 0 & 0 \end{bmatrix}$$

In Eq. (S3), $\boldsymbol{a_1} = a(1,0)$, $\boldsymbol{a_2} = a(-1/2, \sqrt{3}/2)$ and $\boldsymbol{a_3} = a(-1/2, -\sqrt{3}/2)$ are the three primitive vectors. Benefiting from C_{6v} symmetry of our system, we can rewrite the Hamiltonian with another basis $M = \left(|s\rangle, |p_x\rangle, |p_y\rangle, |d_{x^2-y^2}\rangle, |d_{xy}\rangle, |f\rangle\right)^T$, $\alpha = \uparrow\downarrow$, with

$$\begin{aligned} |s\rangle &= [1\quad 1\quad 1\quad 1\quad 1\quad 1]^T/\sqrt{6} \\ |p_x\rangle &= [2\quad 1\quad -1\quad -2\quad -1\quad 1]^T/\sqrt{12} \\ |p_y\rangle &= [0\quad -1\quad -1\quad 0\quad 1\quad 1]^T/2 \\ |d_{x^2-y^2}\rangle &= [2\quad -1\quad -1\quad 2\quad -1\quad -1]^T/\sqrt{12} \\ |d_{2xy}\rangle &= [0\quad -1\quad 1\quad 0\quad -1\quad 1]^T/2 \\ |f\rangle &= [1\quad -1\quad 1\quad -1\quad 1\quad -1]^T/\sqrt{6} \end{aligned} \tag{S4}$$

The Hamiltonian can be transformed to

$$H_1 = \begin{pmatrix} H_{\uparrow\uparrow 1} & H_{\uparrow\downarrow 1} \\ H_{\uparrow\downarrow 1}^{\dagger} & H_{\downarrow\downarrow 1} \end{pmatrix} = \begin{pmatrix} M^{\dagger}H_{\uparrow\uparrow}M & M^{\dagger}H_{\uparrow\downarrow}M \\ M^{\dagger}H_{\uparrow\downarrow}^{\dagger}M & M^{\dagger}H_{\downarrow\downarrow}M \end{pmatrix}. \tag{S5}$$

The band inversion only happens between p and d states, which means $|s\rangle$ and $|f\rangle$ states can be neglected and thus 12×12 matrices H_1 are turned into 8×8 matrices. Further expanding them around Γ point can help us capture the feature of topological phase transitions, then we can get simplified Hamiltonian H_2

$$H_{\alpha\alpha 2}=\begin{bmatrix} \Delta_\alpha & 0 & i\frac{k_x t_{o\alpha}}{2} & i\frac{k_y t_{o\alpha}}{2} \\ 0 & \Delta_\alpha & -i\frac{k_y t_{o\alpha}}{2} & i\frac{k_x t_{o\alpha}}{2} \\ -i\frac{k_x t_{o\alpha}}{2} & i\frac{k_y t_{o\alpha}}{2} & -\Delta_\alpha & 0 \\ -i\frac{k_y t_{o\alpha}}{2} & -i\frac{k_x t_{o\alpha}}{2} & 0 & -\Delta_\alpha \end{bmatrix}$$

$$H_{\uparrow\downarrow 2}=\begin{bmatrix} \frac{\lambda}{2} & \frac{\sqrt{3}\lambda}{2} & -i\frac{\sqrt{3}k_y\lambda}{6} & i\frac{\sqrt{3}k_x\lambda}{6} \\ -\frac{\sqrt{3}\lambda}{2} & \frac{\lambda}{2} & -i\frac{\sqrt{3}k_x\lambda}{6} & -i\frac{\sqrt{3}k_y\lambda}{6} \\ -i\frac{\sqrt{3}k_y\lambda}{6} & -i\frac{\sqrt{3}k_x\lambda}{6} & \frac{\lambda}{2} & -\frac{\sqrt{3}\lambda}{2} \\ i\frac{\sqrt{3}k_x\lambda}{6} & -i\frac{\sqrt{3}k_y\lambda}{6} & \frac{\sqrt{3}\lambda}{2} & \frac{\lambda}{2} \end{bmatrix} \quad \text{(S6)}$$

$$H_2=\begin{pmatrix} H_{\uparrow\uparrow 2} & H_{\uparrow\downarrow 2} \\ H_{\uparrow\downarrow 2}^{\dagger} & H_{\downarrow\downarrow 2} \end{pmatrix}$$

, where (k,ϕ) is the momentum in polar coordinate, and $\Delta_\alpha = t_{o\alpha} - t_{i\alpha}$.

The topological phase transition can be analyzed at Γ point by setting $k=0$, and the Hamiltonian can be block diagonalized with the basis $(|p_+\rangle_\uparrow, |p_+\rangle_\downarrow, |d_+\rangle_\uparrow, |d_+\rangle_\downarrow, |p_-\rangle_\uparrow, |p_-\rangle_\downarrow, |d_-\rangle_\uparrow, |d_-\rangle_\downarrow)^T$, with $|p_\pm\rangle \equiv |p_x\rangle \pm i|p_y\rangle, |d_\pm\rangle \equiv |d_{x^2-y^2}\rangle \pm i|d_{xy}\rangle$

$$H_\Gamma=\begin{bmatrix} H_{p+} & 0 & 0 & 0 \\ 0 & H_{d+} & 0 & 0 \\ 0 & 0 & H_{p-} & 0 \\ 0 & 0 & 0 & H_{d-} \end{bmatrix}$$

$$H_{p\pm}=\begin{bmatrix} \Delta_\uparrow & \frac{\lambda(\pm\sqrt{3}i+1)}{2} \\ \frac{\lambda(\mp\sqrt{3}i+1)}{2} & \Delta_\downarrow \end{bmatrix} \quad \text{(S7)}$$

$$H_{d\pm}=\begin{bmatrix} -\Delta_\uparrow & \frac{\lambda(\mp\sqrt{3}i+1)}{2} \\ \frac{\lambda(\pm\sqrt{3}i+1)}{2} & -\Delta_\downarrow \end{bmatrix}$$

It means that modes in layer $\uparrow$ and $\downarrow$ are combined. We can find that the product of off-diagonal terms in $H_{p,d+}$ is the same with that of $H_{p,d-}$, meaning that $\pm$ states are degenerated.

In general case, $\lambda \neq 0$, and the Hamiltonian can be solved in detail:

$$E_{p1}=\frac{(\Delta_\uparrow+\Delta_\downarrow)+\sqrt{(\Delta_\uparrow+\Delta_\downarrow)^2-4(\Delta_\uparrow\Delta_\downarrow-\lambda^2)}}{2}$$
$$\text{with } |p\rangle_{\pm 1}=\left(\sin\frac{\theta}{2}\right)|p\rangle_{\pm\uparrow}+e^{\mp\frac{\pi}{3}i}\left(-\cos\frac{\theta}{2}\right)|p\rangle_{\pm\downarrow} \quad \text{(S8)}$$

$$E_{p2} = \frac{(\Delta_\uparrow + \Delta_\downarrow) - \sqrt{(\Delta_\uparrow + \Delta_\downarrow)^2 - 4(\Delta_\uparrow\Delta_\downarrow - \lambda^2)}}{2}$$

$$\text{with } |p\rangle_{\pm 2} = \left(\cos\frac{\theta}{2}\right)|p\rangle_{\pm\uparrow} + e^{\mp\frac{\pi}{3}i}\left(\sin\frac{\theta}{2}\right)|p\rangle_{\pm\downarrow}$$

$$E_{d1} = \frac{-(\Delta_\uparrow + \Delta_\downarrow) + \sqrt{(\Delta_\uparrow + \Delta_\downarrow)^2 - 4(\Delta_\uparrow\Delta_\downarrow - \lambda^2)}}{2}$$

$$\text{with } |d\rangle_{\pm 1} = \left(\sin\frac{\theta}{2}\right)|d\rangle_{\pm\uparrow} + e^{\mp\frac{2\pi}{3}i}\left(-\cos\frac{\theta}{2}\right)|d\rangle_{\pm\downarrow}$$

$$E_{d2} = \frac{-(\Delta_\uparrow + \Delta_\downarrow) - \sqrt{(\Delta_\uparrow + \Delta_\downarrow)^2 - 4(\Delta_\uparrow\Delta_\downarrow - \lambda^2)}}{2}$$

$$\text{with } |d\rangle_{\pm 2} = \left(\cos\frac{\theta}{2}\right)|d\rangle_{\pm\uparrow} + e^{\mp\frac{2\pi}{3}i}\left(\sin\frac{\theta}{2}\right)|d\rangle_{\pm\downarrow}$$

, where

$$\theta = \text{acot}\frac{(\Delta_\uparrow - \Delta_\downarrow)}{2\lambda} \in (0, \pi). \quad \text{(S9)}$$

Degeneracy between these four values happens when

$$\lambda^2 = \Delta_\uparrow\Delta_\downarrow. \quad \text{(S10)}$$

The degenerate eigenvalues are

$$\begin{aligned} E_{p1} = E_{d2} = 0, \text{if } \Delta_{\uparrow\downarrow} < 0 \\ E_{p2} = E_{d1} = 0, \text{if } \Delta_{\uparrow\downarrow} > 0 \end{aligned} \quad \text{(S11)}$$

, which matches the phase transition.

The factors $\exp(\mp\pi i/3)$ and $\exp(\mp 2\pi i/3)$ in the eigenstates can be taken as anticlockwise rotation operators $\hat{C}_{6z}$ according to Group theory. In the condition $\Delta_\uparrow = \Delta_\downarrow$, $\theta = \pi/2$, leading to $\sin(\theta/2) = \cos(\theta/2) = 1/\sqrt{2}$, which makes the eigenstates more concise: $|p,d\rangle_{\pm M} = \left(|p,d\rangle_{\pm\uparrow} + (-1)^M \hat{C}_{6z}|p,d\rangle_{\pm\downarrow}\right)/\sqrt{2}$. Equally, we can take them as $|\psi\rangle_M = \left(|\psi\rangle_\uparrow + (-1)^M \hat{C}_{6z}|\psi\rangle_\downarrow\right)/\sqrt{2}$, in which $|\psi\rangle = |p_x\rangle, |p_y\rangle, |d_{x^2-y^2}\rangle$, or $|d_{xy}\rangle$.

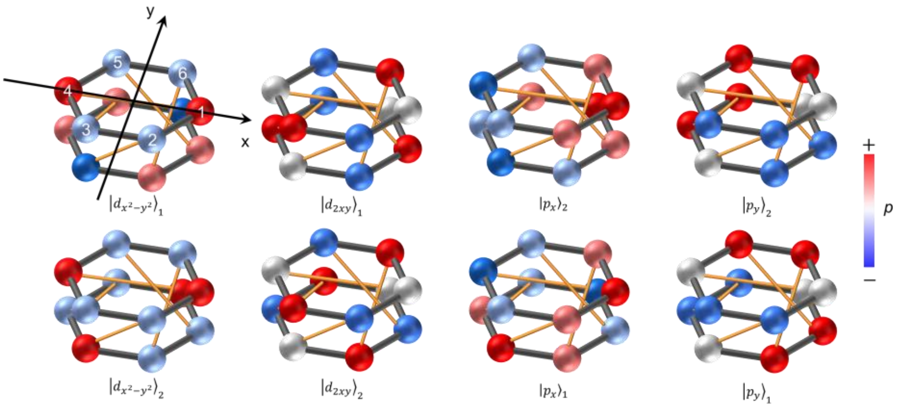

Fig. S6 | Eigen states at Γ point. $|\psi\rangle_M = \left(|\psi\rangle_\uparrow + (-1)^M \hat{C}_6|\psi\rangle_\downarrow\right)/\sqrt{2}$.

II. Pseudo time-reversal symmetry $\hat{C}_{2y}K$

With the basis $\left(|p_x\rangle_2 + i|p_y\rangle_2, |d_{x^2-y^2}\rangle_2 + i|d_{xy}\rangle_2, |p_x\rangle_1 - i|p_y\rangle_1, |d_{x^2-y^2}\rangle_1 - i|d_{xy}\rangle_1, i|p_x\rangle_2 + |p_y\rangle_2, i|d_{x^2-y^2}\rangle_2 + |d_{xy}\rangle_2, -i|p_x\rangle_1 + |p_y\rangle_1, -i|d_{x^2-y^2}\rangle_1 + |d_{xy}\rangle_1\right)^T/\sqrt{2}$, the Hamiltonian with wave vector $\vec{k}$ can be rewritten as

$$H = \begin{bmatrix} \Delta+\lambda & A_1k_- & 0 & 0 & 0 & 0 & 0 & S^*k_- \\ A_1^*k_+ & -\Delta-\lambda & 0 & 0 & 0 & 0 & -Sk_+ & 0 \\ 0 & 0 & \Delta-\lambda & A_2k_+ & 0 & -Sk_+ & 0 & 0 \\ 0 & 0 & A_2^*k_- & -\Delta+\lambda & S^*k_- & 0 & 0 & 0 \\ 0 & 0 & 0 & Sk_+ & \Delta+\lambda & -A_1^*k_+ & 0 & 0 \\ 0 & 0 & -S^*k_- & 0 & -A_1k_- & -\Delta-\lambda & 0 & 0 \\ 0 & -S^*k_- & 0 & 0 & 0 & 0 & \Delta-\lambda & A_2^*k_- \\ Sk_+ & 0 & 0 & 0 & 0 & 0 & -A_2k_+ & -\Delta+\lambda \end{bmatrix} \tag{S12}$$

, in which $k_\pm = k_x \pm ik_y$, $S = e^{-i2\pi/3}(2+\Delta)/8$, $A_1 = \sqrt{3}e^{i2\pi/3}(2+\Delta+4\lambda/3)/8$, $A_2 = \sqrt{3}e^{i\pi/3}(2+\Delta-4\lambda/3)/8$.

The $\hat{C}_{2y}$ operator has the form $-\sigma_y \otimes I_4$, and the pseudospin time-reversal symmetry is $T_f = \hat{C}_{2y}K$. It can be checked that $[T_f, H] = 0$, and $T_f^2 = -1$, which means that Kramers degeneracy can exist in the system. What's more important, this symmetry $\hat{C}_{2y}$ always holds on the boundary, implying that there is no mini-gap in the boundary band and it is insensitive to the boundary condition.

III. Spin-Chern number

In classical wave systems, the spin-Chern number can be calculated by projecting the lower energy bands into spin space with the help of proper spin operator. With the basis $\left(|p_x\rangle_2 + i|p_y\rangle_2, |d_{x^2-y^2}\rangle_2 + i|d_{xy}\rangle_2, |p_x\rangle_1 - i|p_y\rangle_1, |d_{x^2-y^2}\rangle_1 - i|d_{xy}\rangle_1, i|p_x\rangle_2 + |p_y\rangle_2, i|d_{x^2-y^2}\rangle_2 + |d_{xy}\rangle_2, -i|p_x\rangle_1 + |p_y\rangle_1, -i|d_{x^2-y^2}\rangle_1 + |d_{xy}\rangle_1\right)^T/\sqrt{2}$, the spin operator can be taken as its original form $\sigma_z \otimes I_4$. Considering the transformation of basis, the spin operator is considered as $S = U^\dagger(\sigma_z \otimes I_6)U$ for the original Hamiltonian H with 12 bands, in which

$$U = \frac{1}{2\sqrt{3}}\begin{bmatrix} \sqrt{2} & -\sqrt{2} & \sqrt{2} & -\sqrt{2} & \sqrt{2} & -\sqrt{2} & 0 & 0 & 0 & 0 & 0 & 0 \\ \sqrt{2} & \sqrt{2} & \sqrt{2} & \sqrt{2} & \sqrt{2} & \sqrt{2} & 0 & 0 & 0 & 0 & 0 & 0 \\ 1 & e^{-i\theta} & e^{-i2\theta} & -1 & e^{i2\theta} & e^{i\theta} & e^{-i\theta} & e^{-i2\theta} & -1 & e^{i2\theta} & e^{i\theta} & 1 \\ 1 & e^{-i2\theta} & e^{i2\theta} & 1 & e^{-i2\theta} & e^{i2\theta} & e^{-i2\theta} & e^{i2\theta} & 1 & e^{-i2\theta} & e^{i2\theta} & 1 \\ 1 & e^{i\theta} & e^{i2\theta} & -1 & e^{-i2\theta} & e^{-i\theta} & e^{-i2\theta} & e^{-i\theta} & 1 & e^{i\theta} & e^{i2\theta} & -1 \\ 1 & e^{i2\theta} & e^{-i2\theta} & 1 & e^{i2\theta} & e^{-i2\theta} & e^{-i\theta} & e^{i\theta} & -1 & e^{-i\theta} & e^{i\theta} & -1 \\ 0 & 0 & 0 & 0 & 0 & 0 & \sqrt{2} & -\sqrt{2} & \sqrt{2} & -\sqrt{2} & \sqrt{2} & -\sqrt{2} \\ 0 & 0 & 0 & 0 & 0 & 0 & \sqrt{2} & \sqrt{2} & \sqrt{2} & \sqrt{2} & \sqrt{2} & \sqrt{2} \\ i & e^{i\varphi} & e^{-i\varphi} & -i & -e^{i\varphi} & -e^{-i\varphi} & e^{i\varphi} & e^{-i\varphi} & -i & -e^{i\varphi} & -e^{-i\varphi} & i \\ i & e^{-i\varphi} & -e^{i\varphi} & i & e^{-i\varphi} & -e^{i\varphi} & e^{-i\varphi} & -e^{i\varphi} & i & e^{-i\varphi} & -e^{i\varphi} & i \\ -i & e^{-i\varphi} & e^{i\varphi} & i & -e^{-i\varphi} & -e^{i\varphi} & -e^{-i\varphi} & -e^{i\varphi} & -i & e^{-i\varphi} & e^{i\varphi} & i \\ -i & e^{i\varphi} & -e^{-i\varphi} & -i & e^{i\varphi} & -e^{-i\varphi} & -e^{i\varphi} & e^{-i\varphi} & i & -e^{i\varphi} & e^{-i\varphi} & i \end{bmatrix} \qquad \text{(S13)}$$

$\theta = \pi/3,\ \varphi = 5\pi/6.$

Following the similar procedure introduced in Ref [1], we can obtain the gapped spin spectrum and spin-projected Berry curvature for trivial, IQSH, and SHO phases, as shown in Fig. S7. Herein, a rectangle Brillouin zone (BZ) $\{k_x \in [-\pi/a, \pi/a], k_y \in [-2\pi/\sqrt{3}a, 2\pi/\sqrt{3}a]\}$ was used for simplicity and was discretized into a grid size of 161×201 points. Note that both the spin-up and spin-down sectors are composed of three spin bands, and the spin-Chern number is the composite Chern number of three bands as well. By integrating the Berry curvature, we get $C_\pm = \pm 1$ when $|\lambda| > |\Delta|$ in the IQSH phase and $C_\pm = 0$ when $|\lambda| < |\Delta|$ in the trivial and SHO phases because of the balance of positive and negative Berry curvature, which is consistent with the existence of gapless helical edge in IQSH phases.

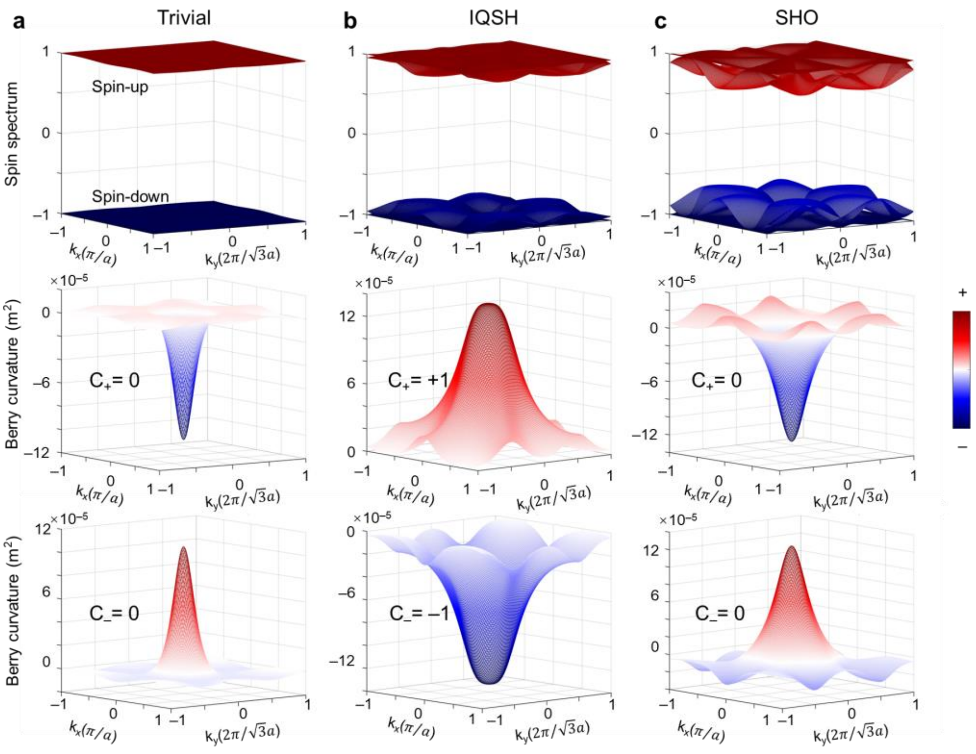

Fig. S7 | Spin spectrum and spin-Chern number for trivial, IQSH and SHO phases. In the calculations λ is set to $1/\sqrt{3}$ while $\Delta = -1/\sqrt{3} - 0.4$ for **a** trivial case, $\Delta = 1/\sqrt{3} - 0.4$ for **b** IQSH case and $\Delta = 1/\sqrt{3} + 0.4$ for **c** SHO case.

IV. Robustness of helical edge states

As long as the T_f symmetry is kept, edge states in the IQSH phase are robust against different kinds of disorders, including Z-shaped corners and site deformation & cavity. The site deformation is realized by increasing the size of sites by 0.5 times. The boundary contains about 10 UCs in both samples. The signal is input on the left side, and the measured transmission spectra can be got from the output on the right side. One can see samples and the transmission spectrum in experiment in Fig. S8. No matter what kinds of disorders are placed, the edge states transmission is lossless.

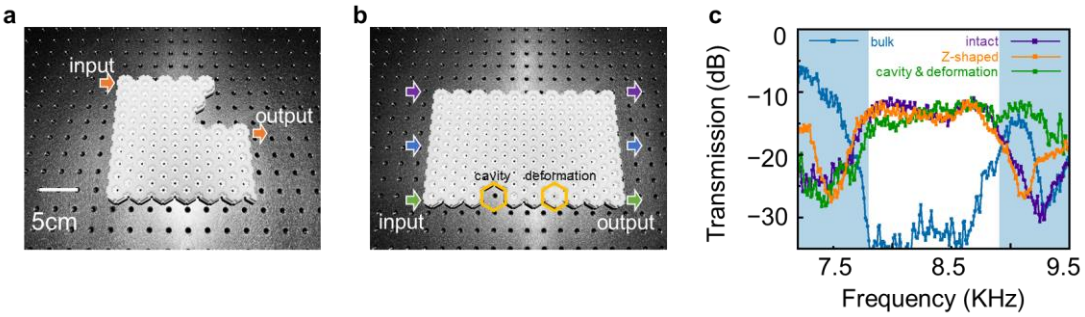

Fig. S8 | Robustness of edge states in IQSH phase against different kinds of disorders. a. The sample with Z-shaped corners. **b.** The sample with site-deformation and cavity. **c.** Transmission spectrum.

V. Winding helical edge states

Based on the design of once-winding topological slow wave with an additionally pendant cavity on the boundary, we can further slowdown the sound velocity by adding and tuning cavities ($R_c = 0.96a/6, 0.195a/6$) with tubes ($w_o = 1\text{cm}, 0.09\text{cm}$) as pedants. The dispersion winds twice and is flattened more significantly (Fig. S9). The maximum tiny gap is reduced to 0.1% relative to the gap center and 0.8% relative to the gap width.

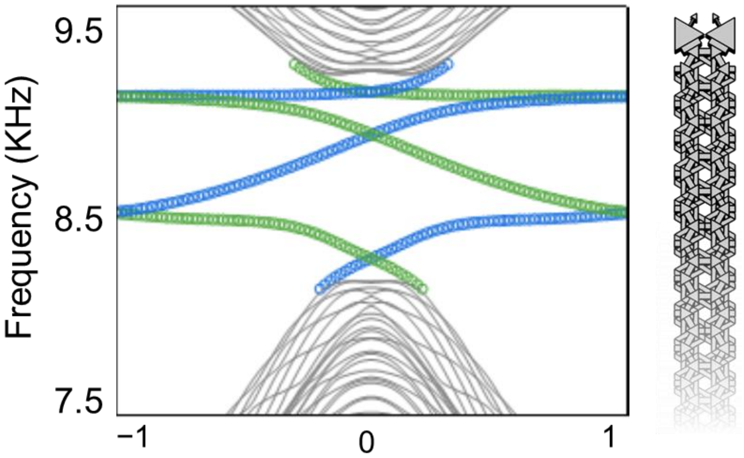

Fig. S9 | Twice-winding acoustic helical edge dispersions. The simulated dispersion winds twice with two deformed pendant cavities on each boundary site.

While for previous acoustic topological states[1-3], the robust winding is hard to be realized due to the lack of a global T_f at the Brillouin zone's center and boundary. This flaw of T_f will be enlarged and harshly break the edge dispersion when meeting resonators. Thus, the simplest design of pedants will destroy the spin-momentum locking and robust wave propagation, leading to backscattering. (Fig. S10).

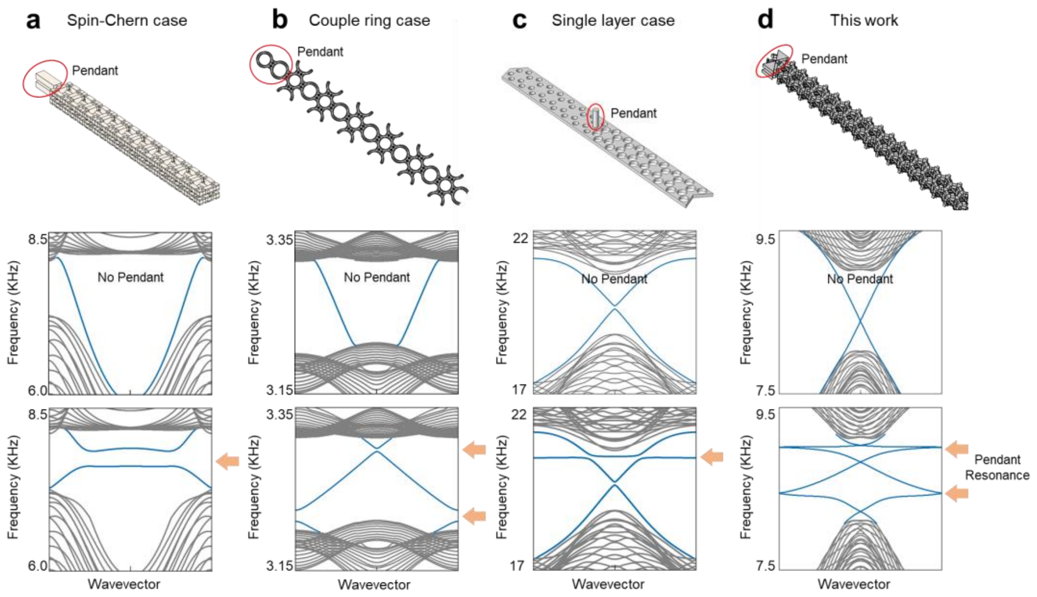

Fig. S10 | Various acoustic topological waveguides coupling with local resonators (pendants). **a**. Acoustic spin-Chern case[1]. **b**. Coupled ring resonator network case[2]. **c**. Single-layer QSH case[3]. **d**. This work under winding twice. Orange arrows denote the resonating frequencies.

Dispersion winding can be confirmed by TBA. As shown in Fig. S11a, two kinds of boundaries are considered here. The typical UCs are shown in blue and green frames, respectively, in which boundary 1 is the one in the manuscript. For convenience, we make $\Delta_{\uparrow\downarrow} = 0$, leading to $t_{\mathrm{o}\uparrow\downarrow} = t_{\mathrm{o}} = t_{\mathrm{i}\uparrow\downarrow} = t_{\mathrm{i}} = 1$ represented by solid and lines. The arrow line represents inter-layer couplings $\lambda = 0.58$. The pendant cavities are coupled to boundaries by simply adding sites coupling to boundary sites. The coupling strengths are set to be t_1, t_2 and t_3 from close to far. For boundaries 1 and 2, $t_{1,2,3} = -0.60, -0.20, -0.12;\ -1.0, -0.30, -0.20$, and the corresponding dispersions are shown in the upper and lower panels of Fig. S11b. It should be noticed that there remain tiny gaps at the center and boundary of BZ. The maximum value is about 0.9% and 0.2% for boundaries 1 and 2, respectively, relative to the bulk gap.

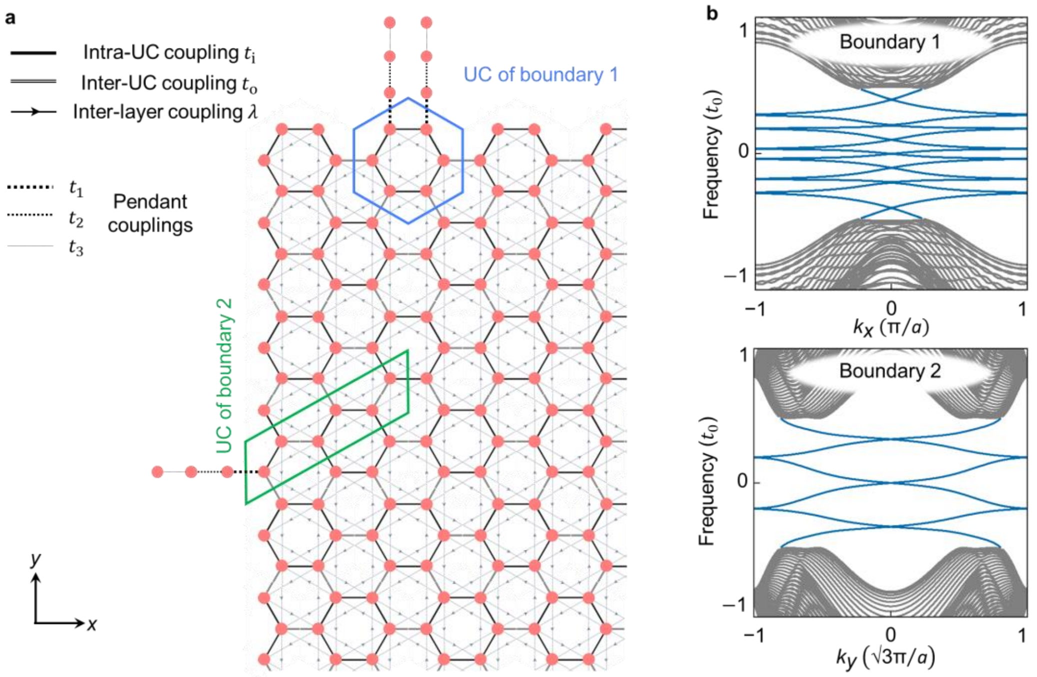

Fig. S11 | Dispersion winding based on TBA. a. The configuration of two kinds of boundaries. Pendant resonators connect to the sample directly. **b.** Dispersion windings of two kinds of boundaries.

VI. Fractional corner charges

Because layer components are coupled through inter-layer coupling, the expression for $Q_c^{\uparrow\downarrow}$ should contain the weight of each layer $\uparrow\downarrow$ and be expressed as

$$Q_c^{\uparrow\downarrow} = \frac{1}{4}\sum_i(|\langle\uparrow\downarrow|\psi_i(M)\rangle|^2 - |\langle\uparrow\downarrow|\psi_i(\Gamma)\rangle|^2) \bmod 1, \tag{S14}$$

where $|\psi_i(\boldsymbol{k})\rangle$ is the low-energy wave function with $+1$ eigen value of $\hat{C}_{2z}$ operator. For $HO_\uparrow$ phase, the eigen states on M and Γ are shown in Fig. S12, and the eigen values of $\hat{C}_{2z}$ operator considering the weight of layer $\uparrow\downarrow$ are shown under every state. The value ± 1 indicate that the acoustic pressure field is on only one layer, while ± 0.5 indicate that it distributes equally on both layers. Only positive values contribute to the fractional corner charge. It can be checked that for pseudospin $\uparrow$, there is only 1 state that mainly contributes +1 value on *M*, while 4 states that mainly contribute +3 value on Γ, which leads to fractional charge approximately $Q_c^{\uparrow} = 1/2$. The situation of pseudospin $\downarrow$ is different, but the total corner charge can be found as $Q_c = Q_c^{\uparrow} + Q_c^{\downarrow} = 1/2$, indicating only one corner state. In fact, the eigen values of $\hat{C}_{2z}$ are not exactly equal to 0, ± 1 or ± 0.5, since the interplay of two layers, *e.g.*, the 3rd and 4th states in line 3, always exists even extremely weak. This deviation makes fractional charge $Q_c^{\uparrow\downarrow}$ away from $1/2$ or 0. But their sum remains quantized $Q_c = Q_c^{\uparrow} + Q_c^{\downarrow} = 1/2$.

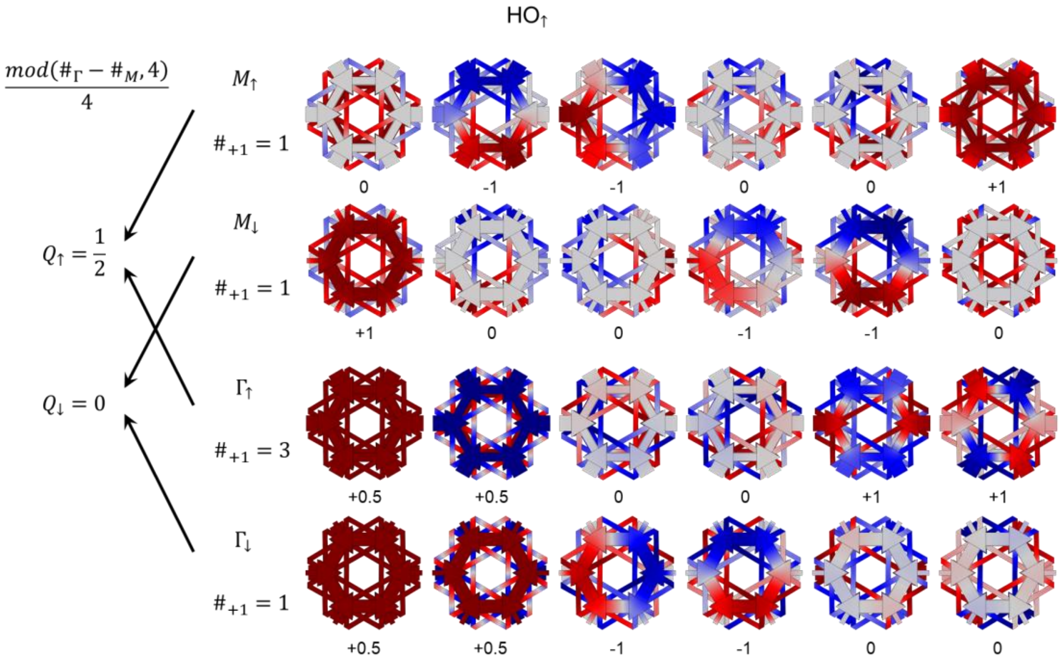

Fig. S12 | Eigen states of pseudospins $\uparrow\downarrow$ on *M* and Γ in $HO_\uparrow$ phase. The eigen values of $\hat{C}_{2z}$ operator considering the weight of pseudospin $\uparrow\downarrow$ is shown under every state.

As for the situation of SHO, $Q_c^{\uparrow} = Q_c^{\downarrow} = 1/2$ can be checked in the Fig. S13, which implies that there are two corner states as what is demonstrated in the manuscript.

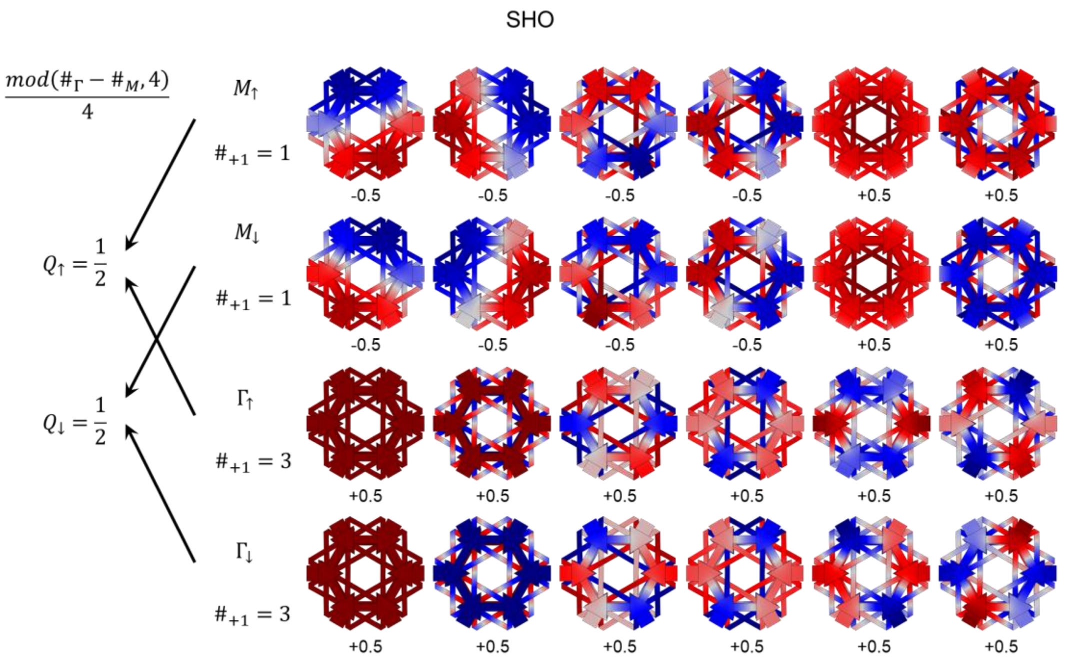

Fig. S13 | Eigen states of pseudospins $\uparrow\downarrow$ on *M* and Γ in SHO phase. The eigen values of $\hat{C}_{2z}$ operator considering the weight of pseudospin $\uparrow\downarrow$ is shown under every state.

References

1 Deng, W. *et al.* Acoustic spin-Chern insulator induced by synthetic spin–orbit coupling with spin conservation breaking. *Nat.Commun.* **11**, 3227 (2020).

2 Wei, Q., Tian, Y., Zuo, S.-Y., Cheng, Y. & Liu, X.-J. Experimental demonstration of topologically protected efficient sound propagation in an acoustic waveguide network. *Phys. Rev. B* **95**, 094305 (2017).

3 He, C. *et al.* Acoustic topological insulator and robust one-way sound transport. *Nat. Phys.* **12**, 1124-1129 (2016).
